# Evidence of TB Services at Primary Healthcare Level during COVID-19: A Scoping Review

**DOI:** 10.3390/diagnostics11122221

**Published:** 2021-11-27

**Authors:** Thobeka Dlangalala, Alfred Musekiwa, Alecia Brits, Kuhlula Maluleke, Ziningi Nobuhle Jaya, Kabelo Kgarosi, Tivani Mashamba-Thompson

**Affiliations:** 1 School of Health Systems and Public Health, Faculty of Health Sciences, University of Pretoria, Pretoria 0001, South Africa; alfred.musekiwa@up.ac.za (A.M.); u15266304@tuks.co.za (K.M.); jaya.nobuhle@mut.ac.za (Z.N.J.); 2 School of Medicine, Faculty of Health Sciences, University of Pretoria, Pretoria 0001, South Africa; u19118296@tuks.co.za; 3 Department of Biomedical Science, Faculty of Natural Science, Mangosuthu University of Technology, KwaZulu-Natal, Umlazi 4031, South Africa; 4 Library Services, Faculty of Health Sciences, University of Pretoria, Pretoria 0001, South Africa; kabelo.kgarosi@up.ac.za; 5 Faculty of Health Sciences, University of Pretoria, Pretoria 0001, South Africa; tivani.mashamba-thompson@up.ac.za

**Keywords:** COVID-19, coronavirus, tuberculosis, health services, primary healthcare

## Abstract

Tuberculosis (TB) is still a major public health concern, despite the availability of preventative and curative therapies. Significant progress has been made in the past decade towards its control. However, the emergence of the novel coronavirus disease 2019 (COVID-19) has disrupted numerous essential health services, including those for TB. This scoping review maps the available evidence on TB services at the primary healthcare (PHC) level during the COVID-19 period. A comprehensive literature search was conducted in PubMed, Web of Science, Medline OVID, Medline EBSCO, and Scopus. A total of 820 articles were retrieved from the databases and 21 met the eligibility criteria and were used for data extraction. The emerging themes were the effect of the COVID-19 pandemic on TB services, patient and provider experiences, recommendations for TB services during the COVID-19 period, and the implementation of the recommendations. The review found that the mitigation strategies, as well as fear and stigma experienced at the start of the COVID-19 pandemic may have led to TB cases potentially going undetected, which may threaten TB treatment outcomes. Therefore, efforts must be directed at finding these missing cases and ensuring that PHC facilities are equipped to adequately diagnose and treat them.

## 1. Introduction

Despite the availability of vaccinations and chemotherapy for prevention and treatment [1], 10 million new cases of tuberculosis (TB) were estimated to have occurred in 2019 [2]. However, only 7.1 million of these cases were found and reported to national TB programmes, leaving a third undetected [3]. In addition, considerably more were not started on an appropriate treatment [1]. These missed cases contribute to the ongoing transmission [4], while prolonged diagnosis and treatment initiation exacerbate disease severity and continued spread [5]. Interrupting transmission through early and accurate detection, rapid treatment initiation, and completion, preferably at the primary healthcare level (PHC), aids efforts in ending the TB epidemic [3,6]. In 2020, COVID-19 emerged, hindering global TB control efforts [7], and sidelining many routine TB services to accommodate the response to the COVID-19 pandemic [8,9]. TB services suffered a sharp decline due to lockdowns. Therefore, limiting access to healthcare and a rise in fear and stigma since the advent of COVID-19 [8,10,11].

Studies that predict the potential impact of the COVID-19 pandemic on TB services suggest that temporary disruptions in response to the pandemic will likely affect all aspects of the TB care cascade [12,13,14]. Even small disruptions to these services could have long-term consequences on TB control [12]. These will especially be felt in high burden countries where TB incidence and mortality have been predicted to increase by 6.3 and 1.4 million, respectively, between 2020 and 2025 [12]. Delays in timely diagnosis and treatment are listed as the potential drivers for these grim outcomes [12,14].

The World Health Organization’s (WHO) End TB strategy and the sustainable development goal (SDG) 3.3 aim to end TB through timely diagnosis and treatment, treatment adherence, and preventative therapy [15,16]. The WHO aims to eliminate the TB epidemic by 2035 and has also set short-term milestones to reduce TB deaths and incidence rates by 2020 and 2025 [3,15]. Findings from the TB global health report showed that 2020 milestones were not achieved [3,17]. Similarly, interim targets were set by the United Nations (UN) to diagnose and treat 40 million additional people by 2022 [7]. Although progress towards these goals has been made, it is still below the threshold that would make TB elimination attainable [3,18]. Moreover, it is possible that the small gains made towards controlling TB were disrupted by the COVID-19 pandemic, pushing the global TB targets further into the future [7,19].

As the first point of contact with health services, PHC facilities can reach large proportions of the population. These facilities also promote equitable access to health services and continuity of care and are recognized as a powerful tool for achieving the health SDGs [16,20]. Moreover, the WHO has emphasized that progress towards containing the TB epidemic can accelerate when TB control has been integrated with PHC [21]. Furthermore, high-quality PHC services are an important predictor for whether TB control strategies will realize their promise [22]. 

Despite the emergence of other public health priorities, such as the COVID-19 pandemic, uninterrupted TB services at the PHC level are crucial for reaching TB targets. Given the novelty of the COVID-19 pandemic, its effects on TB services at the PHC level remain unclear and require further exploration. Therefore, this scoping review mapped evidence on TB services at the PHC level during the COVID-19 pandemic. This evidence will be used to develop the primary research in order to address and improve TB services at the PHC level during the COVID-19 pandemic.

## 2. Materials and Methods

### 2.1. Overview

Herein, we conducted a scoping review to map the available evidence on TB services during the COVID-19 era. This scoping review is conducted as part of a larger study that aims to develop a novel approach for improving TB diagnostic services during the pandemic in primary healthcare clinics in high disease burdened settings. A scoping review protocol was registered on the open science framework (OSF) under the title, “Evidence of TB services at primary healthcare level during COVID-19: A scoping review protocol”, where it can be accessed via this link: https://osf.io/pq3ba, 15 October 2021. The scoping review was guided by the Arksey and O’Malley framework [23], Levac et al. [24], and the Joanna Briggs Institute 2020 guidelines [25]. The findings of the study were reported according to the Preferred Reporting Items for systematic reviews and meta-analyses extension for scoping reviews (PRISMA-ScR) checklist, Table A1 [26].

Step 1: Identifying the research question

The main research question was: What evidence exists on TB services at the PHC level during the COVID-19 pandemic? 

We assessed the eligibility of the research question for a scoping review study by applying the population, concept, and context (PCC) framework, developed by the Joanna Briggs Institute [25], see Table 1. 

Step 2: Identifying relevant studies

We conducted an advanced search using the following five academic databases: PubMed, Web of Science, Medline OVID, Medline EBSCO, and Scopus. Studies were identified using the following keywords and Medical Subject Heading (MeSH) terms: “TB diagnostics”, “Health Service” “TB testing” “COVID-19”, “SARS-CoV-2”, “COVID-19 Pandemic”, “COVID-19 era”, and “Primary healthcare”. A combination of Medical Subject Headings (MeSH) and free word texts of the keywords were used when conducting the searches. WHO and Stop TB partnership websites were accessed for reports and the reference lists of all the included studies were consulted for additional literature. The comprehensive database search was conducted by an experienced librarian to ensure that the best search strategies were used for each database.

Publications that adhere to the following criteria were included:

- Studies reporting on TB services during COVID-19;
- Studies reporting on TB services at PHC;
- All of the publications reporting evidence on TB services during COVID-19 at PHC, regardless of study design;
- Studies from all countries around the world.

This review excluded studies based on the following:

- Studies reporting on TB services outside the PHC level;
- Studies reporting evidence on TB services and viral diseases other than COVID-19;
- Studies reporting evidence on health services other than TB during COVID-19;
- Publications from before 2020.

Step 3: Selecting studies

The studies were selected in three phases. First, the principal investigator screened the titles of each article using the eligibility criteria as a guide. Eligible articles were exported to an EndNote20 library where duplicates were identified and removed. In the second phase, two independent reviewers screened the abstracts of the included articles using a screening tool based on inclusion and exclusion criteria. The screening tool was piloted and adjusted using 10 articles before the screening process was conducted. The reviewers discussed any discrepancies that arose until they reached a consensus on the articles to select. In the third phase, the two reviewers screened the full texts of the relevant articles using a screening tool guided by the eligibility criteria. Before use, the screening tool was piloted by both screeners, and changes were made accordingly. Discrepancies during full-text screening were resolved by a third reviewer. The level of agreement between the two reviewers was calculated using the Kappa statistic. 

Step 4: Charting the data

An electronic data charting form containing variables relevant to the research question was developed. Two independent reviewers then piloted the data extraction tool using 10 of the included studies. The necessary changes were applied according to the feedback given by the reviewers. Data were extracted from the included studies based on the following categories: Author, aim, type of publication, country, type of TB service, and primary healthcare provider.

### 2.2. Quality Appraisal

We determined the methodological quality of the included studies using the Mixed Methods Appraisal Tool (MMAT) V.2018 software [27]. The particular study design used in each article was appraised, following stipulations by the MMAT guidelines. Once the scores for each study were calculated as a percentage, they were given a specific rank. Studies equal to or below 50% were ranked as low quality, those between 51–75% were deemed average quality, and those ranging from 76–100% were given a high-quality score. 

### 2.3. Collating, Summarizing, and Reporting Results

We employed the thematic analysis to extract relevant evidence to answer our research questions and presented a narrative summary that centered on the emerging themes. The themes that arose most from the included studies were as follows: The consequences of the COVID-19 pandemic on TB services, comparison of TB services before and after the COVID-19 pandemic, patient experiences of TB services during COVID-19, and recommendations for TB services at PHCs during COVID-19.

## 3. Results 

### 3.1. Screening Results

**Figure 1:**
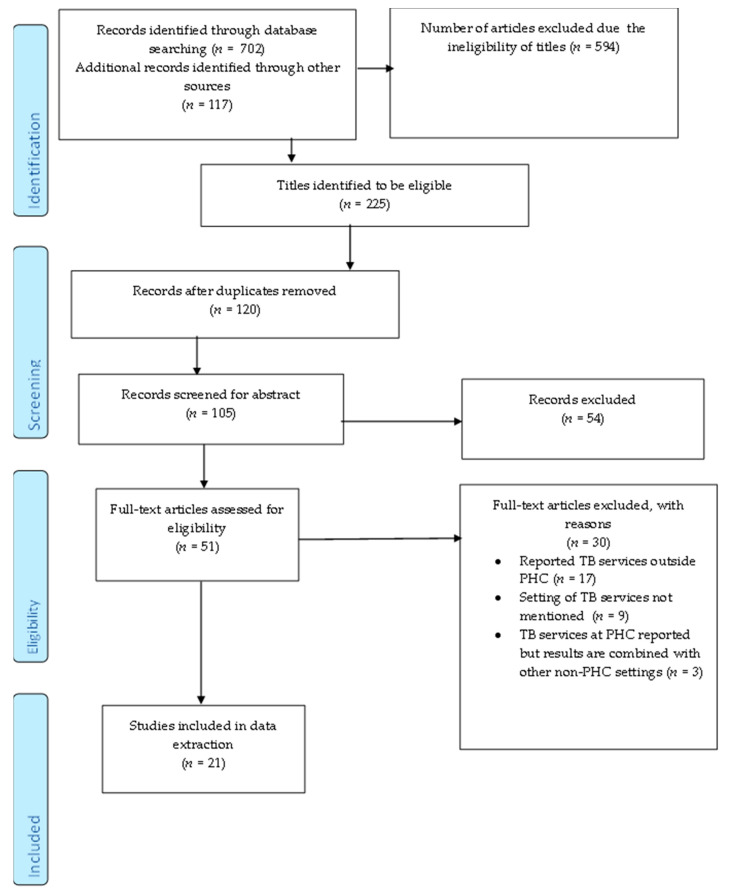
Prisma-flow diagram depicting the process of selecting and excluding studies.

The selection and exclusion of studies are depicted in the PRISMA-ScR flow chart (Figure 1). Initially, we retrieved 819 articles, 702 from database searches and 117 from Google. Following title screening, we excluded 594 ineligible articles. The 225 remaining articles were imported to Endnote 20. The results retrieved from each database are listed in Table 2. After removing 120 duplicates, 105 articles were eligible for abstract screening. A total of 54 articles were excluded after abstract screening and 51 were eligible for full-text screening. We excluded 30 articles after full-text screening. All of the articles reported findings from the pandemic and articles were excluded if they reported TB services outside of PHC (17), did not mention healthcare setting (9), and combined data on TB services from both PHC and higher healthcare settings (3). In total, 21 articles met the eligibility criteria and were used for data extraction. The responses of the reviewers had a 54.64% agreement versus a 73.77% expected agreement by chance, which equates to a moderate agreement (Kappa statistic = 0.4218, *p*-value < 0.05). The discrepancies from the full-text screening were resolved by a third screener.

### 3.2. Characteristics of the Included Studies

The characteristics of the included articles are detailed in Table 3. The studies presented evidence on TB services at the PHC level during the COVID-19 era. The findings were conveyed in a variety of formats including letters, editorials, expert opinion, reports, webinars, feature articles, news articles, and traditional research articles. In terms of countries, the included articles were from Portugal [28], Ethiopia [29], Japan [30], China [9], Malawi [31], the United States of America [32], Pakistan [33,34], Nigeria [35,36,37], India [38,39,40], South Africa [41,42,43], one provided recommendations for high burdened settings [44], one presented evidence from LMIC [45], and one study was addressed to all the countries [46]. The primary healthcare settings ranged from clinics, outpatient departments, general practitioner’s practices, PHC centers, and pharmacies. 

### 3.3. Quality Appraisal 

Only four articles were primary studies presenting empirical evidence and were subject to a methodological quality assessment using the 2018 version of the MMAT tool [27]. The scores ranged from 40–75%. Two studies scored 60% [9,32] and another scored 40% [47] and 70% [35]. Results that scored lower than 51% were considered low quality, 51–75% were of average quality, and high quality if they fell between 76–100%.

### 3.4. Summary of the Evidence

The themes that emerged from the included studies were, consequences of COVID-19 pandemic on TB services, patient and provider experiences, recommendations and adaptations for TB services during the COVID-19 era, and implementing the recommendations for TB services, respectively.

#### 3.4.1. Consequences of the COVID-19 Pandemic on TB Services

Of the 21 included studies, 10 reported on the consequences of the COVID-19 pandemic at various PHC facilities. TB clinics in New York, USA temporarily halted the performance of any new TB tests [32]. A study from a LMIC reported that fewer TB cases were diagnosed due to the difficulty in accessing primary care [45], while a clinic in Nigeria reported that one person came to collect the TB medication during the lockdown [37]. South Africa experienced a 25% drop in access to primary healthcare following the lockdown, as well as a 9% drop in TB testing [41]. Another study in China reported that 75.3% of primary healthcare workers were reallocated from routine services to COVID-19 related work [9]. In a similar manner, clinics from Ethiopia were repurposed as COVID-19 centers [29] or in the case of TB clinics in New York, USA, closed altogether [32]. In Japan, the media reported a shortage of the BCG vaccine in order to claim that it was effective against COVID-19 [30]. 

A project that brought TB healthcare to the doorstep of a community was abruptly halted after the nationwide lockdown in India [39]. This project was aimed at rendering a neighborhood block TB-free and achieved it by actively finding TB cases and providing point-of-care mobile diagnostic services. The effects were seen by the abrupt drop in TB notifications during the 3 months of the national lockdown. In addition, direct comparisons with the same period from previous years showed a stark contrast. Another study in Nigeria that sought to directly compare TB case notifications and detection rates in the first few months of 2020 compared with the same period from 2019 showed similar results [35]. Another study from Ethiopia showed that patient flow had significantly decreased in the first months of the COVID-19 lockdowns compared with the same period from the previous year [29]. Moreover, TB case notifications at primary healthcare centers in Malawi were shown to be disproportionately lower than at a regional hospital in Malawi [31]. The current evidence shows that the COVID-19 pandemic has created a scenario where fewer TB cases were detected than usual. However, more evidence is required to determine the extent of the potentially missed cases.

#### 3.4.2. Patient and Provider Experiences

Four of the included studies recorded the perspectives of healthcare workers and patients. All of the participants struggled to access healthcare facilities. Rumors on the closure of certain facilities meant that patients were not seeking care for a period of time in Malawi [31]. In India, 17.3% of patients defaulted on their TB treatment and others consulted general practitioners and private pharmacies for treatment due to the difficulty in accessing healthcare facilities [38]. A survey by the Stop TB partnership found that in several countries, fear of contracting COVID-19 kept patients away from visiting clinics [47]. Likewise, in Malawi, fear and ignorance of COVID-19 meant that many healthcare personnel refused to see or treat anyone displaying symptoms resembling COVID-19 [31]. Moreover, staff were increasingly reluctant to handle any sputum samples or observe sputum collection. Furthermore, this was the case in Nigeria [32,38]. A lack of personal protective equipment (PPE) discouraged staff from attending to patients in many countries [31,47]. A survey by the Stop TB partnership found that staff at TB clinics observed a need for patients to be given nutritional support, as well as have their transportation costs covered for visiting healthcare facilities [47]. 

#### 3.4.3. Recommendations and Adaptations of TB Services

Five studies from multiple authors including the WHO have detailed recommendations on how TB services can be improved during a pandemic in high burden settings. All of the studies agreed that the use of telemedicine can be leveraged for TB care. Medical triage and counselling should be conducted by telephone. Where possible, sputum collection should be conducted in a well-ventilated area at home and staff must be adequately protected when collecting the samples from patients [46]. The switch to oral and shorter treatment regimens [42], as well as the video-supported treatment would reduce the number of patients visiting health facilities [46]. Integrating TB and COVD-19 care, such as testing and active case finding, could benefit the management of both diseases [42]. HIV care must also be integrated for countries with a high disease burden [42]. Patients with drug-susceptible TB should be provided with enough TB medication for the intensive phase and only return to the healthcare facility for an assessment. In addition, they need to switch to the continuation phase where sufficient medication is provided [43,45]. Patients with drug-resistant TB (DR-TB) must be switched to an oral treatment that lasts until the next scheduled visit, any patients exhibiting concerning iron levels or myelosuppression must be recalled by telephone [44,46]. Moreover, decentralizing the treatment collection has been encouraged [42,46]. Furthermore, there was an emphasis on strengthening primary care in order to help in managing the pandemic, by providing PHC workers with best practice training for COVID-19 [43,46]. This ensures that PHC facilities are equipped with enough staff who have access to PPE and provision of all chronic medication should be available for extended periods to reduce visits to health facilities [43]. Finally, all of the PHCs offering TB testing must follow the recommended infection prevention and control (IPC) measures, from the collection of samples until testing is conducted and the sample is disposed of in the laboratory [46]. It is not clear how many high burden countries have implemented these changes for their TB programs and how successful implementation has been. The following section explores examples of instances where TB services have been adapted. 

#### 3.4.4. Implementing the Recommendations for TB Services

Five of the included studies documented the changes made to the TB services in response to the COVID-19 pandemic. Outpatient departments in India and Portugal screened patients for COVID-19 before they were attended to. Crowd control was also maintained to ensure that social distance and IPC measures are upheld at all times [28,40]. The same center in Portugal did contact tracing by telephone and patients were only asked to come to the clinic if they had a positive screening after the phone call [28]. The oral treatment is now favored over injectables and treatment is administered in line with scheduled healthcare visits in India and the USA [32,40]. Those requiring intravenous treatments are administered by community nurses at home [40]. In several countries, treatment initiation is conducted in clinics, but all of the follow-ups are conducted by telephone, including any consultation with doctors, unless presenting with severe symptoms or treatment side effects [28,31,34,40]. TB clinics in New York, USA, have also begun giving patients daily reminders over the phone to ensure that they adhere to the treatments [28]. In cases where patients cannot utilize telehealth due to limitations in technology, then home visits are conducted on a case by case basis [32]. In Pakistan, general practitioners (GPs) who referred patients to TB centers were used to locate patients that could not be contacted during the pandemic. Moreover, their offices were used as a location where patients could fetch their medication [33]. Furthermore, certain provinces in Pakistan have mandated that private healthcare providers notify TB cases to national TB programs [34]. Healthcare workers are provided with the necessary PPE according to the risk of exposure and they work in shifts to avoid overcrowding [40]. In Pakistan, healthcare providers have been retrained in IPC and the correct use of PPE wherever necessary [34]. All of these adaptations are new and will need to be closely monitored throughout the pandemic to assess their sustainability and effectiveness. Furthermore, more data are needed on other high burden countries to see whether they have adapted TB services since the start of the pandemic.

## 4. Discussion

This scoping review mapped the existing evidence of TB services at the PHC level in the COVID-19 era. The evidence was from a wide range of documentary sources, and most came from high TB burden regions of Pakistan, India, Nigeria, and South Africa. The bulk of the literature found was from the start of the pandemic. The findings show evidence that the COVID-19 pandemic had a negative effect on TB services, how patients and healthcare providers were impacted, as well as recommendations for adapting these services and instances where recommendations had been implemented. Overall, the COVID-19 pandemic has negatively impacted TB services, users, and healthcare providers alike. The findings suggest that TB services were disrupted. In addition, the fear and stigma experienced by healthcare providers and patients likely led to a drop in TB case detection and the notifications seen during the first months of the pandemic. More evidence is needed on the steps taken to identify potentially undiagnosed and missed TB cases and how provider attitudes and patient experiences have improved, especially in high TB burden countries. Although the review has highlighted recommendations for enhancing TB services in high burden settings during the pandemic, only one TB endemic country had implemented these changes. Before COVID-19, countries were making strides towards achieving the SDG targets for TB; a record number of people had been treated including those with DR-TB; the annual number of missed TB cases had fallen below 3 million; and the TB preventative treatment had been prioritized in high burden settings [7]. However, this has likely changed since the start of the COVID-19 pandemic.

The findings of this scoping review show that the arrival of COVID-19 and the measures used to curb the spread drastically reduced the number of TB cases detected and notified, in sharp contrast to the numbers from the same period in previous years [29,32,36]. It further demonstrated how TB services were significantly disrupted and sidelined in response to a new public health emergency [9,32,37,45]. Moreover, the COVID-19 pandemic deterred health-seeking behaviors, hindered some patients from acquiring the TB treatment, and increased reluctance among healthcare workers to treat patients [31,37]. These results have created scenarios for TB cases to go undiagnosed. Furthermore, the fear of attending health facilities and the disruptions leading to their closure have likely interrupted TB treatment regimens, which could lead to treatment failure exacerbating disease transmission and development of drug resistance. These would be grim outcomes for global TB control efforts. The responses, uncovered by the review, mirror those experienced during the Ebola virus outbreak in West Africa, which increased preventable TB deaths over time [48]. Following the Ebola outbreak, TB, HIV, and malaria deaths exceeded those directly caused by the Ebola virus itself [49,50]. Similarly, the outbreak of the Middle East respiratory syndrome coronavirus (MERS-CoV) in Saudi Arabia harmed TB control efforts [51]. As a result of the Ebola outbreak [52], certain West African countries have implemented measures for epidemic response. However, it is unclear whether these have had any bearing on TB control during the COVID-19 era. The increase in TB mortality in the coming years due to the disruptions to health services has been foreshadowed by several studies [13,14,53]. These will be most evident in high burden settings unless swift action is taken to minimize the impact on health services, while simultaneously identifying, diagnosing, and treating any cases that are not from the start of the pandemic. 

A study summarizing the effect of Ebola on TB services emphasized the importance of moving away from disease-specific national programmes to the holistic strengthening of health systems [48]. It further highlights how this kind of approach would not only assist with the management of infectious outbreaks, but ensure that disease control for other conditions is not compromised [48]. Two previous studies showed that China’s and Saudi Arabia’s prior coronavirus experience facilitated a better COVID-19 response than many other countries [54,55]. In contrast, this review did not find evidence of TB endemic countries adopting the lessons from previous epidemics. However, our findings present current recommendations for conducting TB services during the COVID-19 pandemic [42,43,44,46]. Although these are helpful, it would have been more beneficial if governments had adopted insights from past viral outbreaks. The review also demonstrates how the recent adaptations to TB services have been adopted in various countries [28,32,33,34,40]. However, only two of these were high TB burden countries [34,40]. Considering that many of these suggestions rely on the use of technology, their practicality for resource-limited settings remains to be seen. Therefore, high burden countries must continue to monitor the impact of COVID-19 on TB services and address these with evidence-based interventions.

### 4.1. Implication for Research

Many of the included studies documented the situation at the start of the COVID-19 pandemic. Consequently, geographic areas with a lower incidence at the start of the pandemic were not a focus of this study. Thus, an assessment of TB services in these regions is needed for better insight into the global effect of the COVID-19 pandemic. Research on how TB services have fared throughout the pandemic, including peaks in COVID-19 cases and subsequent vaccination strategies, are also needed. The same can be said for provider and health seeker attitudes. This will facilitate the measurement of the impact on TB and allow appropriate mitigation action. Undiagnosed TB cases and interruption to treatment were other issues likely to have been caused by the pandemic. Health systems must be ready to receive and appropriately treat and retain these cases until treatment completion. Therefore, assessing the quality of TB services in high burden settings and providing context-specific adaptations based on the findings could benefit TB control programmes. Moreover, only four empirical studies were found and even these scored low in terms of methodological quality. Therefore, robust primary studies are required to inform evidence-based decisions and recommendations for TB services during pandemics. These studies should focus on strengthening TB case findings, diagnostics, and treatment services for COVID-19 and future pandemics.

### 4.2. Strengths and Limitations

The scoping review employed a comprehensive database search that was not limited by language, publication or study design. In addition, the database search included a grey literature and repeated search in the database that retrieved the highest number of articles to maximize the number of studies found. The methodological quality of all the included primary studies was assessed and it was found that they ranged from a low to average quality. For this reason, the scoping review may not be appropriate to inform clinical practice, but does demonstrate a need for additional primary studies to be conducted with more methodological rigor. Moreover, the review retrieved evidence from the start of the COVID-19 pandemic. Therefore, certain geographic regions with initially low incidence rates were not covered. Furthermore, given the evolving nature of the pandemic, it is likely that latter phases including the emerging variants and vaccination control strategies have also impacted the TB service delivery.

## 5. Conclusions

In this review, the TB services at the PHC level were disrupted by the COVID-19 pandemic. The potential for undiagnosed TB cases and treatment failure are among the biggest concerns caused by the pandemic. For the TB elimination goals to be met, PHC must be strengthened and ready with effective solutions to address the issues caused by the COVID-19 pandemic and use these for pandemic preparedness in the future.

## Figures and Tables

**Table 1:** PCC framework to determine the eligibility of the research question and guide the selection of studies on TB services during the COVID-19 pandemic.

Determinants	Description
Population	Primary healthcare providers—healthcare practitioners providing TB services, which are the first point of contact between people in a community and the healthcare system.
Concept	TB services—the processes involved in finding, diagnosing, treating, and preventing TB, which leads to cases being notified to national health systems.
Context	COVID-19 era—the time since COVID-19 emerged, from January 2020 to date.

**Table 2:** Results of the database search.

Date	Database	Keywords	Number of Results Retrieved
7 June 2021	PubMed	((“Health Services” [Mesh] OR “primary health care” [MeSH Terms] OR “Primary health care” [Text Word] OR “health care” [Text Word] OR “health service*” [Text Word] OR “Primary healthcare” [Text Word]) AND (“sars-cov-2” [MeSH Terms] OR “covid-19” [MeSH Terms] OR covid [Text Word] OR coronavirus OR “corona virus”)) AND (“tuberculosis” [MeSH Terms] OR tuberculosis [Text Word])	191
7 June 2021	PubMed	((“primary health care” [MeSH Terms] OR “Primary health care” [Text Word] OR “Primary healthcare” [Text Word]) AND (“sars-cov-2” [MeSH Terms] OR “covid-19” [MeSH Terms] OR covid [Text Word] OR coronavirus OR “corona virus”)) AND (“tuberculosis” [MeSH Terms] OR tuberculosis [Text Word])	13
11 June 2021	Web of Science	(TITLE-ABS-KEY (tuberculosis OR tb) AND TITLE-ABS-KEY (sars-cov-2 OR covid-19 OR covid OR coronavirus OR“corona AND virus”) AND TITLE-ABS-KEY (“primary health care” OR “primary AND healthcare” OR “primary AND care” OR “Health Services”))	5
7 June 2021	Medline OVID	(((MH “COVID-19”)) OR “covid-19” OR ((MH “SARS-CoV-2”)) OR “sars-cov-2”) AND (((MH “Tuberculosis+”)) OR “tuberculosis”) AND (((MH “Primary Health Care”)) OR (“primary health care”) OR ((MH “Health Services+”)) OR (“health services”) OR (“primary health”))	223
7 June 2021	Medline EBSCO	(((MH “COVID-19”)) OR “covid-19” OR ((MH “SARS-CoV-2”)) OR “sars-cov-2”) AND (((MH “Tuberculosis+”)) OR “tuberculosis”) AND (((MH “Primary Health Care”)) OR (“primary health care”) OR ((MH “Health Services+”)) OR (“health services”) OR (“primary health”))	189
7 June 2021	Scopus	(TITLE-ABS-KEY (tuberculosis OR tb) AND TITLE-ABS-KEY (sars-cov-2 OR covid-19 OR covid OR coronavirus OR “corona AND virus”) AND TITLE-ABS-KEY (“primary health care” OR “primary AND healthcare” OR “primary AND care” OR “Health Services”))	81

**Table 3:** Characteristics of the included studies.

Author and Date	Aim of Study	Publication Type	Country	Primary Healthcare Provider	Type of TB Service(s) Reported
Fatima et al. 2021 [34]	To demonstrate how TB services were strengthened during COVID-19	Research article	Pakistan	PHC centers, private healthcare providers (PHCP)	General TB services and case notifications
Aguiar 2021 [28]	To show the changes made at a TB outpatient center as a result of COVID-19	Letter	Portugal	Outpatient center	TB case finding and treatment
Beyene et al. 2021 [29]	To assess the impact of COVID-19 on TB control programs at various clinics in Addis Ababa	Research article	Ethiopia	Public health clinics	TB screening and testing
Comella-del-Barrio et al. 2021 [45]	To give an overview of the effects of COVID-19 on TB control	Editorial	Low to middle-income countries (LMIC)	Primary healthcare in general	TB testing
Fei et al. 2020 [9]	To show how COVID-19 has affected TB control in China	Research article	China	Primary healthcare workers and clinics	General TB services
Adewole 2020 [35]	How COVID-19 has impacted TB care in Nigeria	Letter	Nigeria	TB clinic	TB case notification and detection
Burzynsky et al. 2020 [32]	To show how TB services have been adapted for COVID-19 during the closure of non-essential services in New York	Letter	United States of America	TB clinics	TB detection, testing, and treatment
Cox et al. 2021 [44]	To provide recommendations for TB care during COVID-19 in high burden settings	Letter	Countries with a high TB burden	Clinics	TB treatment
Keene et al. 2020 [42]	How TB and HIV services can leverage the COVID-19 pandemic	Expert Opinion	South Africa	Clinics	TB screening, testing, treatment, and detection
Rai and Kumar 2020 [38]	How TB patients were affected by the lockdown in India	Letter	India	Pharmacists, outpatient department, and general practitioners (GP)	TB treatment
World Health Organization 2020 [46]	To give guidance on how TB care should be conducted during COVID-19	Report	All countries	Outpatient centers and primary healthcare workers	TB treatment
Stop TB partnership 2020 [47]	To show how COVID-19 has impacted different TB stakeholders around the world	Report Survey	Global fund implementing countries	Clinics	General TB services
Soko et al. 2021 [31]	To estimate the impact of COVID-19 on TB case notifications	Research Article	Malawi	Primary healthcare centers	TB case notifications
Meneguim et al. 2020 [40]	How a TB center adapted its service for COVID-19 in India	Letter	India	Outpatient hospital department	TB diagnostics, treatment, follow-up, and adherence support
Pilane et al. 2020 [41]	Reporting disruption of TB and HIV services due to COVID-19	News Article	South Africa	PHC facilities	General TB services
Datta et al. 2020 [40]	To show how COVID-19 disrupted a TB free block model pilot study	Report	India	Mobile diagnostic services	Active case-finding and TB diagnostics
Debriche Health and Development Center 2020 [36]	To discuss how TB and PHC services have been impacted by COVID-19 and propose solutions	Webinar	Nigeria	PHC centers	General TB services
Adepoju 2020 [37]	To demonstrate how COVID-19 has affected TB care	Feature	Nigeria	PHC centers and clinics	TB screening and treatment
Jamal et al. 2020 [33]	To detail how TB services were maintained in the private sector during COVID-19	Letter	Pakistan	GPs	TB treatment and diagnostics
Ongole et al. 2020 [43]	To give insight into how TB care can be conducted during COVID-19 through strengthened PHC	Letter	South Africa	PHC centers	General TB services at PHC
Senoo et al. 2020 [30]	To report on the shortages of the BCG vaccine	Letter	Japan	Clinics	TB vaccinations

## Data Availability

The data for the scoping review was obtained through secondary data analysis, all data supporting the conclusions of this scoping review are available through the reference list.

## Appendix A

**Table A1:** Preferred reporting items for systematic reviews and meta-analyses extensions for scoping reviews (PRISMA-ScR) checklist.

Section and Topic	Item No.	Checklist Item	Reported on Page
ADMINISTRATIVE INFORMATION			
Title:			
Identification	1	Evidence of TB services at the primary healthcare level during COVID-19: A scoping review.	1
Registration	2	Open Science Framework: https://osf.io/pq3ba (accessed on 16 September 2021)	
**INTRODUCTION**			
Rationale	3	Despite the availability of vaccinations and chemotherapy for prevention and treatment [1], 10 million new cases of tuberculosis (TB) were recorded in 2019 [2]. A third of these cases were missed by health systems [3], and considerably more were not started on an appropriate treatment [1]. These missed cases contribute to the ongoing transmission [4], while prolonged diagnosis and treatment initiation exacerbate disease severity and continued spread [5]. Interrupting transmission through early and accurate detection, rapid treatment initiation, and completion, preferably at the primary healthcare level (PHC), aids efforts in ending the TB epidemic [3,6]. In 2020, COVID-19 emerged, hindering global TB control efforts [7], many routine TB services were sidelined in response to the COVID-19 pandemic [8,9]. These services suffered a sharp decline due to lockdowns limiting access to healthcare and a rise in fear and stigma since the advent of COVID-19 [8,10,11]. Studies that predict the potential impact of COVID-19 on TB services suggest that temporary disruptions in response to COVID-19 will likely affect all aspects of the TB care cascade [12,13,14]. Even small disruptions to these services could have long-term consequences on TB control [12]. These will especially be felt by high burden countries where TB incidence and mortality have been predicted to increase by 6.3 and 1.4 million between 2020–2025, respectively [12]. Delays in patients seeking timely diagnosis and treatment are listed as the potential drivers for these grim outcomes [12,14]. The World Health Organization’s (WHO) End TB strategy and the sustainable development goal (SDGs) 3.3 aim to end TB through timely diagnosis and treatment, treatment adherence, and preventative therapy [15,16]. The WHO aims to eliminate the TB epidemic by 2035 and has also set short-term milestones to reduce TB deaths and incidence rates by 2020 and 2025 [3,15]. Findings from the TB global health report showed that 2020 milestones were not achieved [3]. Similarly, interim targets were set by the United Nations (UN) to diagnose and treat 40 million additional people by 2022 [7]. Although progress towards these goals has been made, it is below the threshold that would make TB elimination attainable [3,18]. It is also possible that the small gains made towards controlling TB were disrupted by the COVID-19 pandemic, pushing the global TB targets further into the future [7,19]. As the first point of contact with health services, PHC can reach large proportions of the population. It also promotes equitable access to health services and continuity of care and is thereby recognized as a powerful way that health SDGs can be achieved [16,20]. The WHO has also emphasized that progress towards containing the TB epidemic can accelerate when TB control has been integrated with PHC [21]. Furthermore, high-quality PHC services are an important predictor for whether TB control strategies will realize their promise [22]. Despite the emergence of other public health priorities, such as the COVID-19 pandemic, uninterrupted TB services at PHC are crucial for TB targets to be reached. Given the novelty of the COVID-19 pandemic, its effects on TB services at the PHC level remain unclear and require further exploration. Therefore, this review aimed to systematically map evidence on TB services at the PHC level during the COVID-19 pandemic. The evidence obtained from the study will be used to develop primary research that is aimed at addressing and improving TB services at the PHC level during the COVID-19 pandemic to accelerate global efforts to end TB.	3–4
Objectives	4	This review aimed to systematically map evidence on TB services at the PHC level during the COVID-19 pandemic.	4
**METHODS**			
Eligibility criteria	5	**Inclusion criteria** Publications that adhere to the following criteria were included: Studies reporting on TB services during COVID-19; Studies reporting on TB services at PHC. All of the publications reporting evidence on TB services during COVID-19 at PHC, regardless of study design Studies from all of the countries around the world. **Exclusion criteria** This review excluded studies based on the following: Studies reporting on TB services outside the PHC level; Studies reporting evidence on TB services and viral diseases other than COVID-19; Studies reporting evidence on health services other than TB during COVID-19; Publications from before 2020.	6
Information sources	6	We conducted an advanced search using the following five academic databases: PubMed, Web of Science, Medline OVID, Medline EBSCO, and Scopus.	5
Search strategy	7	Studies were identified using the following keywords and medical subject heading (MeSH) terms: “TB diagnostics”, “Health Service” “TB testing” “COVID-19”, “SARS-CoV-2”, “COVID-19 Pandemic”, “COVID-19 era” and “Primary healthcare”. A combination of medical subject headings (MeSH) and free word texts of the keywords were used when conducting the searches. WHO and Stop TB partnership websites were accessed for reports and the reference lists of all the included studies were consulted for additional literature.	5–6
Study records:			
Data management	8a	Describe the mechanism(s) that will be used to manage records and data throughout the review.	
Selection process	8b	The studies were selected in three phases. First, the principal investigator screened the titles of each article using the eligibility criteria as a guide. Eligible articles were exported to an EndNote20 library where duplicates were identified and removed. In the second phase, two independent reviewers screened the abstracts of the included articles using a screening tool developed through the use of the inclusion and exclusion criteria. The screening tool was piloted and adjusted using 10 articles before the screening process was conducted. The reviewers discussed any discrepancies that arose until they reached a consensus on the articles to select. In the third phase, the two reviewers screened the full texts of the relevant articles using a screening tool guided by eligibility criteria. Before use, the screening tool was piloted by both screeners, and changes were made accordingly. Discrepancies during full-text screening were resolved by a third reviewer. The level of agreement between the two reviewers was calculated using McNemar’s Chi-square statistic.	6
Data collection process	8c	An electronic data charting form containing variables relevant to the research question was developed. Two independent reviewers then piloted the data extraction tool using 10 of the included studies. The necessary changes were applied according to the feedback given by the reviewers.	7
Data items	9	Data were extracted from the included studies based on the following categories: Author, aim, type of publication, country, type of TB service, and primary healthcare provider.	7
Data synthesis	10	We employed thematic analysis to extract relevant evidence to answer our research questions and presented a narrative summary that centered around the emerging themes. The themes that arose most from the included studies were as follows: The unintended consequences of COVID-19 on TB services; comparison of TB services before and after COVID-19; patient experiences of TB services during COVID-19; and recommendations for TB services at PHC during COVID-19.	7
		
		
		
Confidence in cumulative evidence	11	To assess the risk of bias we determined the quality of the included studies using the mixed methods appraisal tool (MMAT) V.2018 software [27]. The tool assessed the methodological quality of the included primary studies. The particular study design guided how the article was appraised, following stipulations by the MMAT guidelines. Once the scores for each study were calculated as a percentage, they were given a specific rank. Studies equal to or below 50% were ranked as low quality, those between 51–75% were deemed average quality, and those ranging from 76–100% were given a high-quality score.	7
